# *SLC38A4* as a prognostic biomarker and correlated with immune infiltration in colorectal liver metastasis

**DOI:** 10.1007/s12672-025-03509-9

**Published:** 2025-09-02

**Authors:** Ying Liu, Xin Wei, Ningning Chen, Weijia Wang

**Affiliations:** 1 Department of Medical Oncology, Shaanxi Provincial People’s Hospital, No. 256 Youyi West Road, Xi’an, 710068 People’s Republic of China; 2 Department of Medical Oncology, First Affiliated Hospital, Xi’an Jiaotong University, Xi’an, China

**Keywords:** SLC38A4, Prognostic biomarker, Colorectal liver metastasis, Immune infiltration, Metabolic reprogramming.

## Abstract

**Background:**

Colorectal liver metastasis (CRLM) is the most frequent form of metastasis and the main reason for deaths associated with colorectal cancer. However, prognostic biomarkers originating from CRLM tissue remain limited. Additionally, the impact of the metabolism-associated gene SLC38A4 on patients with CRLM remains elusive.

**Methods:**

Metabolism-related differentially expressed genes (MRDEGs) were identified between CRLM and adjacent normal liver (NL) tissues using GEO datasets (GSE38174, GSE41258). The prognostic significance of these MRDEGs in CRLM (GSE159216) was evaluated using Cox regression and Kaplan-Meier survival analyses. The differential expression of SLC38A4 was validated through multiple experiments, including qRT-PCR, Western blotting, and immunohistochemistry. Genes co-expressed with SLC38A4 were identified through a weighted gene co-expression network analysis, and enrichment analyses were conducted by *clusterProfiler*. The link between SLC38A4 and immune infiltration was assessed with the *CIBERSORT* algorithm, while drug sensitivity was analyzed using *oncoPredict*.

**Results:**

SLC38A4 was identified as an independent favorable-prognosis biomarker for CRLM, with significantly lower expression in CRLM compared to NL tissues. Enrichment analyses indicated that SLC38A4-associated genes participate in diverse metabolic processes. Immune infiltration analysis indicated that SLC38A4 expression is linked to the infiltration of immune cells and three immune checkpoint genes: ARG1, EDNRB, and TNFSF4. Additionally, multiple anti-tumor drugs were positively associated with SLC38A4 expression.

**Conclusion:**

Elevated SLC38A4 expression is correlated with a favorable prognosis in CRLM, likely through mechanisms involving metabolic reprogramming and immune infiltration. Thus, SLC38A4 may serve as both a prognostic biomarker and a potential biomarker for future therapeutic investigation, offering new precision medicine options for CRLM patients.

**Supplementary Information:**

The online version contains supplementary material available at 10.1007/s12672-025-03509-9.

## Introduction

Colorectal cancer (CRC) ranks as the second highest cause of cancer-related mortality globally, contributing to 9.3% of all deaths [1]. Colorectal liver metastasis (CRLM)—the most prevalent type of CRC metastasis—is the chief cause of CRC-related mortality [2]. With the progression of CRC, approximately 25–50% of diagnosed individuals will develop CRLM, either metachronous or synchronous [3, 4]. Radical resection combined with chemotherapy is still the typical therapy for CRLM [5]. However, surgical intervention is possible for only 10–20% of cases, with a 35% survival rate after five years [6]. The remaining patients ineligible for surgery show a poor prognosis [7]. Therefore, targeting liver metastasis is crucial for enhancing the therapeutic outcomes of patients with CRC by identifying potential biomarkers and novel therapeutic targets.

Biomarkers are central to cancer management, including differential diagnosis, prediction, and assessment of treatment response in CRC [8]. However, limited studies have identified prognostic biomarkers for CRLM. Previous studies have demonstrated that mutations in genes, including *RAS*, *BRAF*, *TP53*, and *SMAD4*, as well as protein biomarkers like THBS1, are predictors of poor prognosis in CRLM [9, 10]. Bioinformatics has facilitated the identification of potential tumor biomarkers, primarily through gene differential expression analysis, hub gene screening, and regulatory network construction, such as miRNA-mRNA networks [11–14]. Additionally, some studies have identified prognostic biomarkers for CRLM, such as *GAS1*, *IMPA2*, *C5*, *CDH2*, and *SPARCL1* [13, 15–17]. However, most of these biomarkers are derived from CRC tissues, which do not accurately reflect the heterogeneity of CRLM [18]. HER2 amplification in liver metastasis tissues is linked to a worse prognosis in individuals with wild-type RAS/RAF genes and left-sided CRLM [19]. Decreased CDX2 expression and elevated CD73 expression in liver metastatic lesions predict a poor prognosis [20, 21]. However, these prognostic biomarkers derived specifically from CRLM tissues remain scarce. This necessitates finding optimal biomarkers specific to CRLM tissues to enhance the efficacy of targeted therapies.

Solute carrier family 38 member 4 (*SLC38A4*)—also known as sodium-coupled neutral amino acid transporter 4—belongs to the amino acid transporter system A. This Na+-dependent transporter mediates the uptake of neutral amino acids [22], which are essential for the proliferation of rapidly dividing cells, particularly cancer cells [23]. However, studies on *SLC38A4* in cancer are sparse. *SLC38A4* can inhibit hepatocellular carcinoma (HCC) progression [24, 25]. Nevertheless, its role in other cancers, particularly liver metastatic cancers, including CRLM, remains unclear. Therefore, the investigation focused on determining the prognostic significance of *SLC38A4* in CRLM and its impact on metabolic and immune functions.

In the research, we explored the metabolism-related differentially expressed genes (MRDEGs) in CRLM tissues compared to nearby normal liver (NL) tissues. *SLC38A4* was found to be a favorable prognostic biomarker and a tumor suppressor gene in CRLM. Furthermore, the weighted gene co-expression network analysis (WGCNA) method was applied to find *SLC38A4*-associated genes, followed by functional enrichment analyses. Finally, immune cell infiltration and drug sensitivity investigations demonstrated the role of *SLC38A4* in CRLM. Taken together, *SLC38A4* might be a novel prognostic biomarker and a potential candidate for future therapeutic investigation.

## Method

### Public database

The Gene Expression Omnibus (GEO) database provided three datasets: GSE38174, GSE41258, and GSE159216. GSE38174 and GSE41258 included gene expression profiles of 30 and 9 paired CRLM and NL tissues, respectively, which were used to identify MRDEGs. GSE159216 encompassed gene expression profiles and clinical information on CRLM tissues. To ensure reliable analysis, samples lacking complete survival data were excluded, and this dataset was designated as the CRLM cohort.

### Identifying MRDEGs

In GSE38174 and GSE41258, the *Limma* package identified differentially expressed genes (DEGs) with thresholds of |log2FC| > 2 and adjusted *p*-values < 0.05. The results were then visualized through Volcano plots. Metabolism-related genes (MRGs), which encode human metabolic enzymes and transporters, were sourced from previous studies [26, 27]. The intersection of DEGs and MRGs yielded MRDEGs, which were analyzed in the CRLM cohort.

### Prognostic and survival analyses

In the CRLM cohort, the expression values of MRDEGs were integrated with clinicopathological variables, including sex, type of liver metastasis, liver resection margin status, extrahepatic metastasis, *KRAS*, *NRAS*, and *TP53* mutation status. The prognostic impact of these MRDEGs was then determined through Cox regression analysis. Subsequently, the Kaplan-Meier approach, along with a log-rank test, was employed to further evaluate their effects on survival outcomes. The *survival* and *survminer* R packages were used, with statistical significance indicated by a *p*-value below 0.05.

### Differential expression analysis of SLC38A4

The transcriptional expression of *SLC38A4* in paired CRLM and NL tissues was analyzed using datasets GSE38174 and GSE41258, along with real-time quantitative reverse transcription PCR (qRT-PCR). Meanwhile, the translational expression of *SLC38A4* was validated through Western blotting and immunohistochemistry (IHC) using in-house tissue samples.

### SLC38A4-related modules and genes screened by WGCNA

Co-expression modules related to *SLC38A4* were identified through WGCNA. These modules were then examined for their correlation with CRLM phenotype. The most significant module was selected for further analysis. Hub genes were identified based on their high gene significance and module membership, with both metrics exceeding 0.5.

### Functional enrichment analyses

Through the *clusterProfiler* package, Gene Ontology (GO) and the Kyoto Encyclopedia of Genes and Genomes (KEGG) pathways were carried out on co-expressed genes associated with *SLC38A4* [28]. Significance was determined by adjusted *p*-values < 0.05.

Based on the median level of *SLC38A4*, individuals with CRLM were grouped into low- and high-expression categories. Gene set enrichment analysis (GSEA) was then conducted through the *clusterProfiler* [28], with statistical significance considered for a false discovery rate below 0.05.

### Immune infiltration analysis

Utilizing the *CIBERSORT* algorithm [29], the tumor microenvironment (TME) of CRLM was analyzed. It estimated the infiltration levels for 22 distinct types of immune cells. To explore the connection between *SLC38A4* expression and the infiltrates of immune cells, Pearson correlation analyses were then conducted.

Additionally, 60 immune checkpoint genes, with 24 functioning as immune inhibitors and 36 as immune stimulators, were selected from previous research [30]. Pearson correlation analyses assessed the associations between the mentioned genes and *SLC38A4* expression. *P* values less than 0.05 indicated significance, with correlations categorized as moderate or strong (*R* > 0.30) and weak (*R* < 0.30).

### Drug sensitivity analysis

The Genomics of Drug Sensitivity in Cancer database supplied the drug information data [31]. The IC50 values for various drugs were assessed according to *SLC38A4* expression using the *oncoPredict* package [32]. Additionally, Pearson’s tests were utilized to calculate correlation coefficients, with *P* values below 0.05 indicating significance.

### Sample collection

Fresh CRLM and matched NL tissues were collected from eight patients during liver metastasis surgical excision and preserved at -80 °C for later analysis. Additionally, twelve paired CRLM and NL formalin-fixed paraffin-embedded (FFPE) pathological sections were acquired. In line with the Declaration of Helsinki, this study received approval from the Ethics Committee of Shaanxi Provincial People’s Hospital (Approval No. 2023-R156). Each participant gave written informed consent.

### qRT-PCR

Trizol reagent (Invitrogen, USA) was used to extract total RNA from fresh tissue, followed by the synthesis of cDNA from the RNA extracts using a reverse transcription system (Takara, Japan). Gene expression was quantified through qRT-PCR (Takara, Japan), using GAPDH as an internal reference and the 2^-ΔΔCt method for result calculation. The specific primer sequences used were as follows: SLC38A4, forward: 5′-AGGCAGCAATGAGCAGTCAA-3′, reverse: 5′-GGTTCCGGGATGGTGTTCAT-3′; GAPDH, forward: 5′-TCGGAGTCAACGGATTTGGT-3′, reverse: 5′-TTCCCGTTCTCAGCCTTGAC-3′.

### Western blotting

Proteins were extracted from fresh tissues using ice-cold RIPA lysis buffer (Cat# P0013; Beyotime, China). After centrifuging the lysates at 13,000 ×g for 20 min at 4 °C, the supernatant was collected. Subsequently, a BCA Kit (Cat# 23225; Thermo Fisher Scientific, USA) was used to measure the protein concentration. In the Western blotting experiment, 30 µg of protein per lane was denatured at 95 °C for 5 min, followed by separation using SDS-PAGE and transfer to a PVDF membrane. After blocking with 5% non-fat milk in TBST for 2 h at room temperature, the membrane was incubated overnight at 4 °C with the anti-SLC38A4 primary antibody (1:600; Cat# 20857-1-AP; Proteintech, China). After three 10-minute washes with TBST, the membrane was incubated with an HRP-conjugated anti-rabbit IgG secondary antibody (1:10,000; Cat# ab6721; Abcam, UK) for 2 h at room temperature. Protein bands were visualized using ECL reagent (Cat# 34580; Thermo Fisher Scientific). The membrane was then stripped with Restore Western Blot Stripping Buffer (Cat# 21059; Thermo Fisher Scientific) for 15 min at room temperature, washed extensively with TBST, reblocked, and reprobed with anti-GAPDH (1:3000; Cat# ab263962; Abcam) under identical conditions. Band intensities were quantified using ImageJ software and normalized to GAPDH.

### Immunohistochemistry

The pathological sections underwent deparaffinization in xylene, followed by rehydration through ethanol solutions of reducing concentrations. Using EDTA buffer at pH 9.0, antigen retrieval was carried out with microwave heating at 95 °C for 20 min. Endogenous peroxidase activity was inhibited using 3% hydrogen peroxide for 10 min, followed by blocking nonspecific antigens with 10% goat serum (Cat# G9023, Sigma, USA) for 1 h. Subsequently, the sections were treated with an anti-SLC38A4 antibody (1:50, Cat# 20857-1-AP, Proteintech, China) for 2 h at 37 °C. Afterward, a biotin-labeled secondary antibody was applied, and DAB chromogenic development was performed for 5 min. After counterstaining with hematoxylin, the sections were dehydrated through graded alcohols and cleared with xylene, after which the slides were mounted with neutral resin. Images were captured using a light microscope (Leica Microsystems, Germany). The staining intensity was quantitatively analyzed using the IHC Profiler plugin in ImageJ [33], with results categorized into four levels: high positive, positive, low positive, and negative.

### Statistical analysis

The differential expression of SLC38A4 was evaluated at the mRNA level using paired t-tests, while protein levels were assessed through unpaired t-tests for Western blotting and chi-square tests for IHC. Pearson’s test was used to conduct correlational analyses. In determining statistical significance, all analyses used a threshold of *p* < 0.05.

## Results

### Study design

**Fig. 1:**
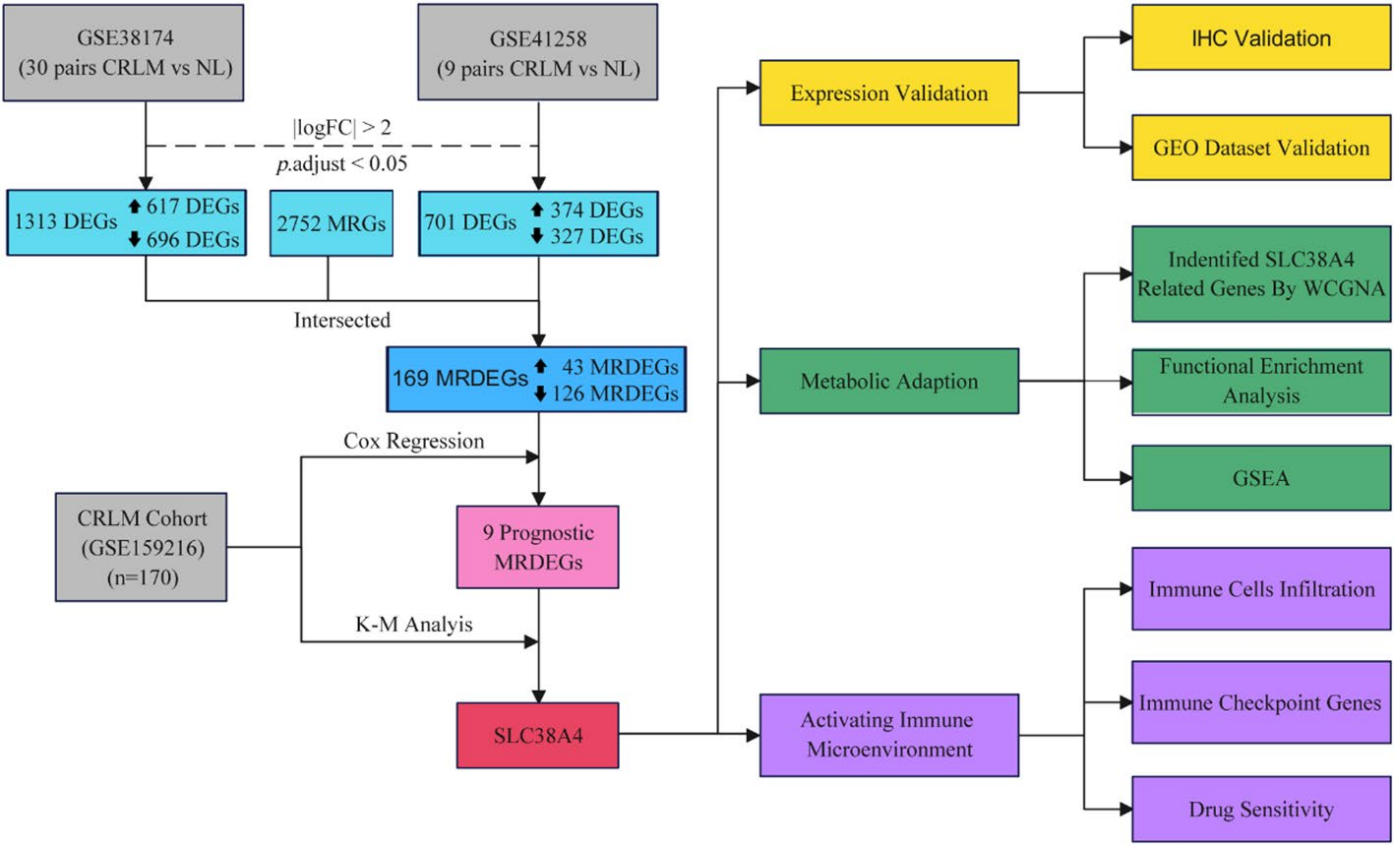
The flow diagram showing the process of this study

Figure 1 illustrates the research process. Initially, the MRDEGs between CRLM and NL tissues were determined by overlapping the DEGs and MRGs. *SLC38A4* was identified as a favorable prognostic biomarker for individuals with CRLM. Furthermore, the differential expression of *SLC38A4* and its potential biological function in CRLM were explored. Lastly, the links between *SLC38A4* expression and immune cell infiltration, as well as drug sensitivity, were investigated.

### Screening MRDEGs between CRLM and NL tissues

**Fig. 2:**
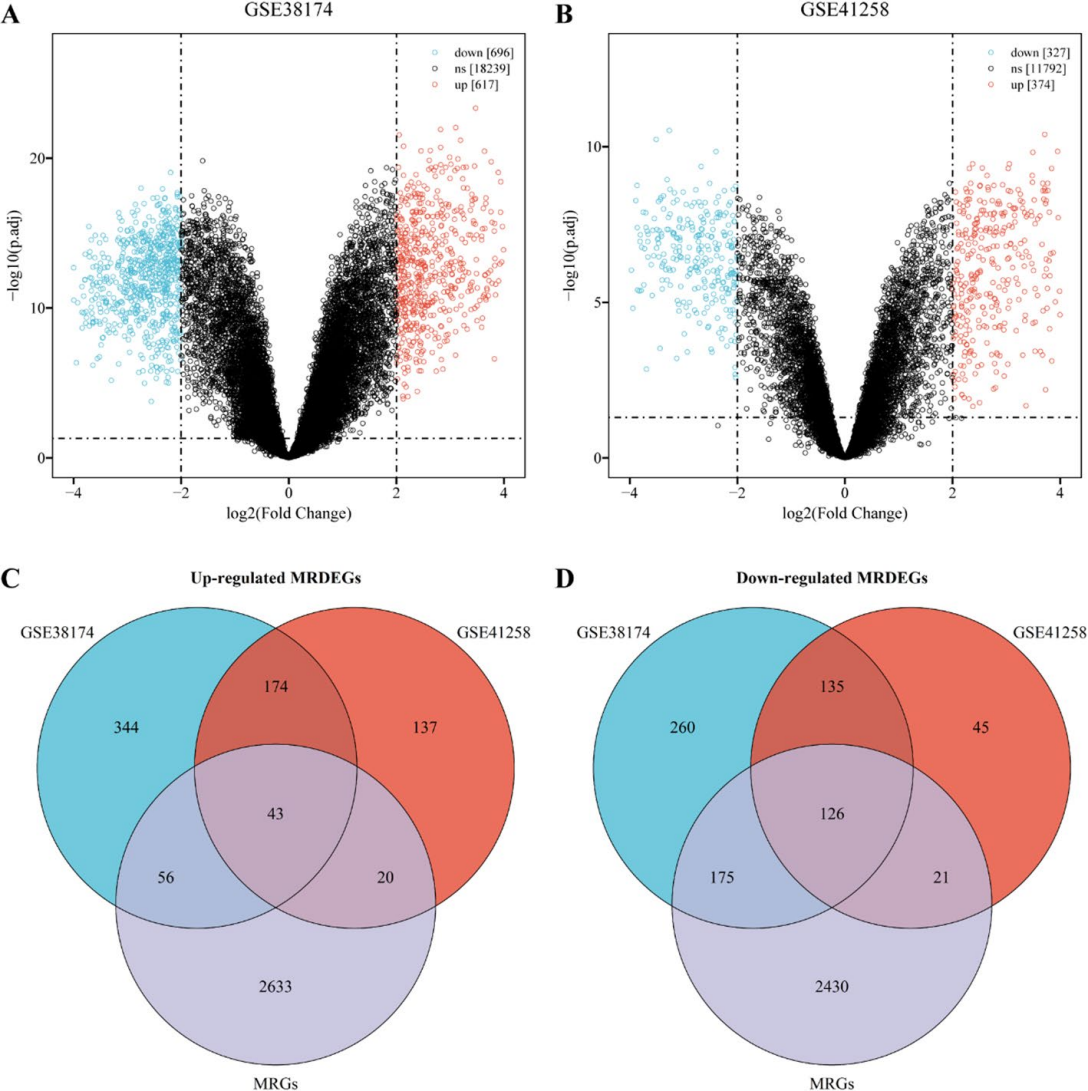
Screening of MRDEGs between CRLM and NL. **A**,** B** Volcano plots for DEGs in GSE38174 and GSE41258; **C**,** D** Upregulated and downregulated MRDEGs generated by the intersection of DEGs and MRGs

Figure 2 presents a comparative analysis of CRLM and paired NL tissues. The dataset GSE38174 included 1,313 DEGs, with 617 genes upregulated and 696 downregulated (Fig. 2A). Similarly, the GSE41258 dataset contained 374 upregulated and 327 downregulated genes (Fig. 2B). For downstream analysis, 2,752 MRGs—encoding human transport proteins and metabolic enzymes—were retrieved from previous studies. The intersection of DEGs and MRGs generated 169 candidate MRDEGs, including 43 upregulated and 126 downregulated MRDEGs (Fig. 2C, D), which were selected for further analysis.

### SLC38A4 is a favorable prognostic biomarker

**Fig. 3:**
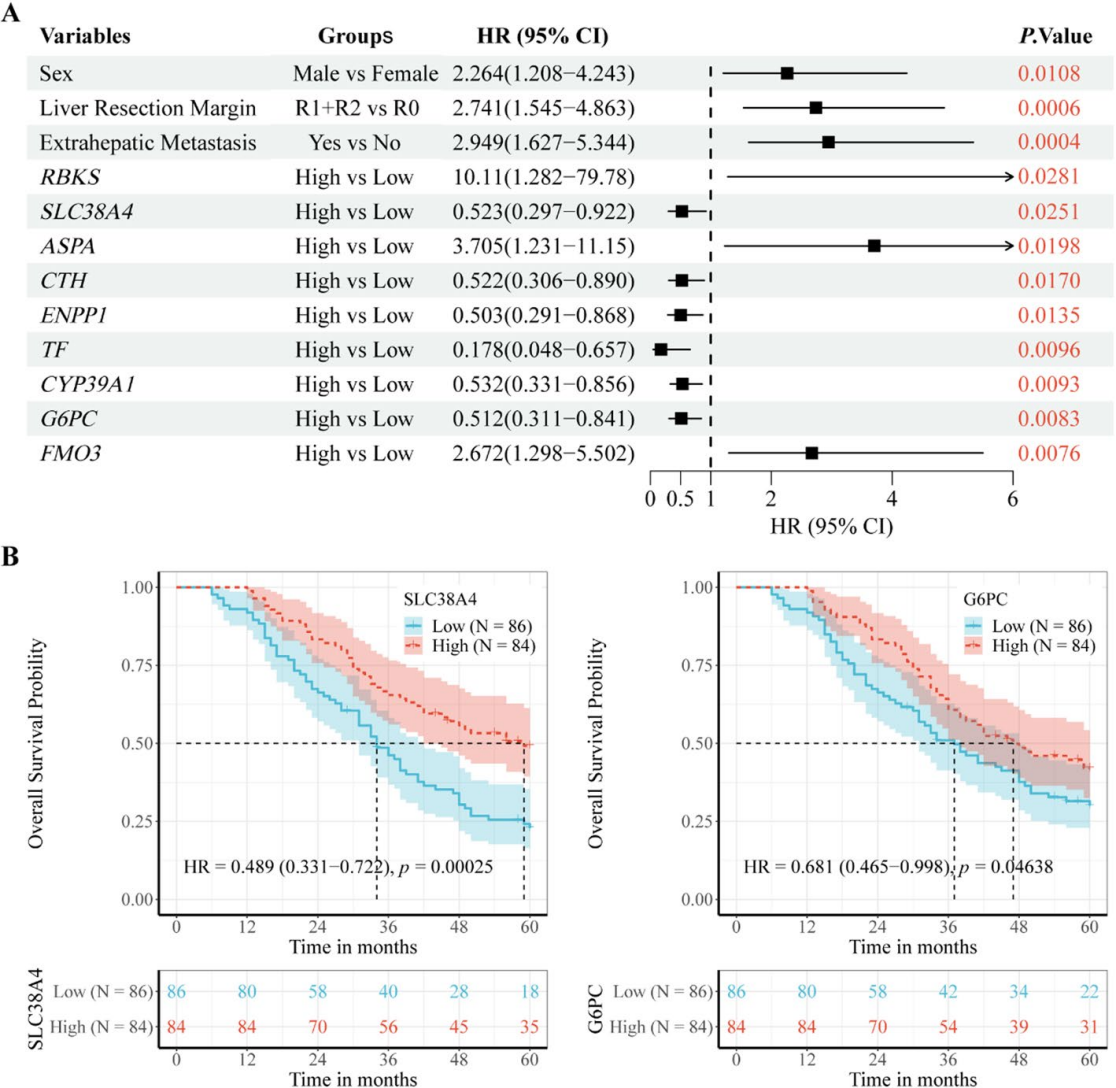
Prognostic implication of MRDEGs in the CRLM cohort. **A** OS-related prognostic MRDEGs identified by multivariate Cox regression analysis; **B** Kaplan-Meier OS curves for prognostic MRDEGs

The prognostic implications of the 169 MRDEGs in the CRLM cohort were evaluated through univariate Cox regression analyses, which identified 48 MRDEGs significantly related to overall survival (OS) (Supplementary Table 1). Further multivariate Cox regression analyses revealed nine independent prognostic MRDEGs, including *SLC38A4*, *RBKS*, *ASPA*, *CTH*, *ENPP1*, *TF*, *CYP39A1*, *G6PC*, and *FOM3* (Fig. 3A). Subsequently, Kaplan-Meier survival analysis stratified patients into low and high expression groups. Notably, only high expression levels of *SLC38A4* (hazard ratio (HR) = 0.489, *p* = 0.00025) and *G6PC* (HR = 0.681, *p* = 0.04638) showed significant connections to OS (Fig. 3B). Compared to *G6PC*, *SLC38A4* may demonstrate a stronger survival benefit, as evidenced by its lower HR. Additionally, *SLC38A4* exhibited higher diagnostic accuracy (Area Under Curve (AUC): 0.953 vs. 0.939) and superior 5-year prognostic prediction performance (AUC: 0.635 vs. 0.584) (Fig. S1). Given its predominant expression in hepatic tissue [34], dysregulation of *SLC38A4* may directly influence the progression of liver metastasis. Based on its relative robust prognostic power, survival prediction performance, diagnostic discrimination, and liver-specific biological relevance, *SLC38A4* was prioritized for further investigation.

### Differential expression of SLC38A4 validated by qRT-PCR, WB, and IHC

**Fig. 4:**
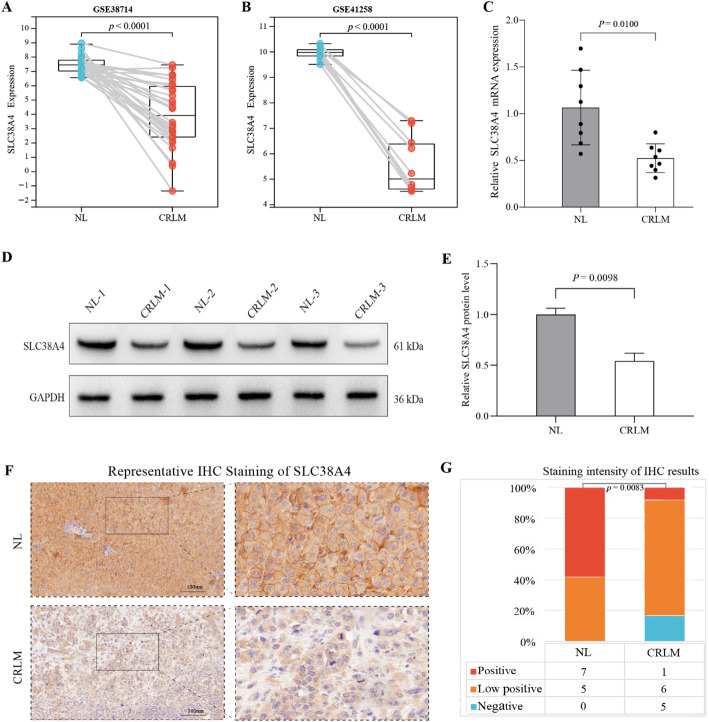
Validation of SLC38A4 differential expression between CRLM and NL.** A**,** B** SLC38A4 mRNA expression in public GEO datasets;** C** qRT-PCR validation in clinical samples (*n* = 8) confirms reduced SLC38A4 expression in CRLM;** D** Representative western blot of SLC38A4 (~ 61 kDa) in matched CRLM and NL pairs;** E** Quantification of western blot signals;** F** Representative IHC staining of SLC38A4 in matched CRLM and NL tissues;** G** Semi-quantitative analysis of IHC staining intensity

At the mRNA expression level, analysis of GEO datasets (GSE38174 and GSE41258) indicated a significant decrease in SLC38A4 expression in CRLM tissues compared to NL tissues (Fig. 4A, B). This finding was further validated in our clinical cohort through qRT-PCR analysis of matched CRLM and NL pairs (*n* = 8), which demonstrated a decrease in SLC38A4 expression in CRLM (*p* = 0.01; Fig. 4C). At the protein level, WB analysis detected a specific SLC38A4 band at the expected molecular weight (~ 61 kDa; Fig. 4D). Quantitative analysis revealed significantly lower SLC38A4 protein expression in CRLM samples compared to NL tissues (*p* = 0.0098; Fig. 4E). IHC examination displayed distinct staining patterns (Fig. 4F). Generally, the IHC staining patterns of SLC38A4 within the tumor were homogeneous. The majority of SLC38A4-positive cells exhibited membranous and uneven cytoplasmic staining, while nuclear staining was rarely observed. These findings align with the subcellular localization information provided by the GeneCards knowledgebase (https://www.genecards.org/) (Fig. S2). Notably, SLC38A4 expression was consistently positive in NL tissues; however, it was downregulated, showing low-positive or negative staining in 91.7% (11 out of 12) of CRLM tissues (Fig. 4G, Supplementary Table 2). Statistical analysis confirmed a significantly reduced SLC38A4 staining intensity in CRLM compared to NL tissues (*p* = 0.0083; Fig. 4G), corroborating both the transcriptomic and WB results.

### Potential function of SLC38A4 in CRLM

**Fig. 5:**
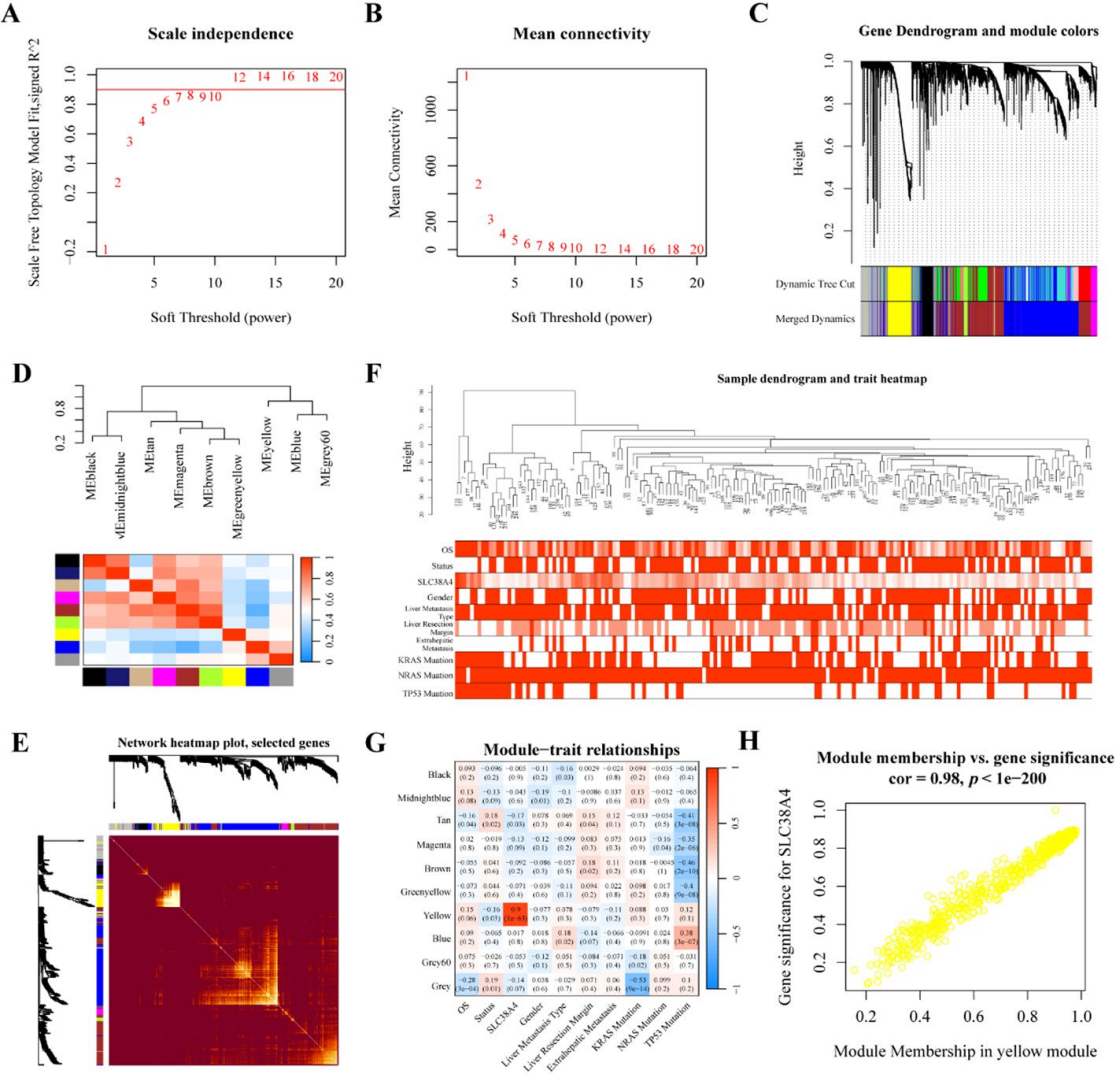
Identification of SLC38A4-associated modules and genes in CRLM. **A**,** B** Assessment of the scale-free fit index and mean connectivity across different soft-thresholding powers (β); **C**–**F** Cluster dendrogram of patients with CRLM, with 10 modules clustered; **G** Correlation heatmap between CRLM phenotype and modules; **H** Scatter plot of the yellow module

WGCNA analysis was conducted to identify *SLC38A4*-related genes and investigate its potential counteractive effects in CRLM progression (Fig. 5A–F). Using average linkage hierarchical clustering, 10 modules were identified from the CRLM cohort. Of these, the yellow module contained 530 genes and demonstrated the strongest association with *SLC38A4* expression (correlation coefficient = 0.98, *p* < 0.0001) (Fig. 5G–H).

**Fig. 6:**
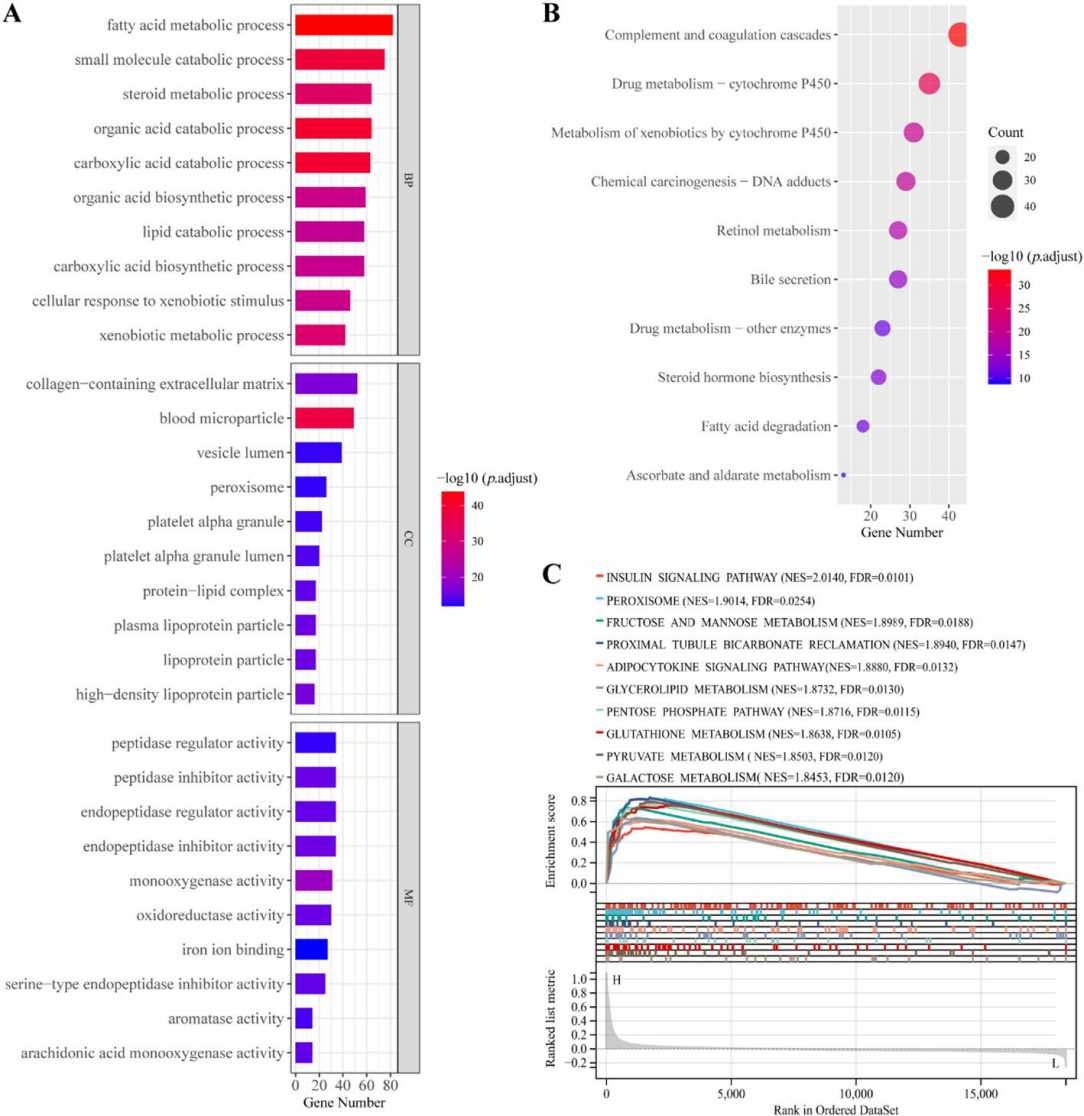
Potential functions of SLC38A4 in CLRM. **A**,** B** Examination of SLC38A4 and its related genes through GO and KEGG; **C** Enriched pathway analysis of the high-expression SLC38A4 group by GSEA

The analysis of GO term annotation for these 530 genes suggested that *SLC38A4* is significantly linked to biological processes, primarily metabolic and catabolic processes (Fig. 6A). Cellular component analysis identified its presence in the collagen-containing extracellular matrix, blood microparticle, and vesicle lumen. Molecular function analysis demonstrated the role of *SLC38A4* in enzymatic activities, such as peptidase, endopeptidase, monooxygenase, and oxidoreductase activities. KEGG pathway analysis elucidated the involvement of *SLC38A4* in drug metabolism, xenobiotic metabolism by cytochrome P450, retinol metabolism, and fatty acid degradation (Fig. 6B).

Additionally, GSEA results suggested that high *SLC38A4* expression is predominantly linked to metabolic pathways, such as glycerolipid, glutathione, pyruvate, galactose, fructose, and mannose metabolism (Fig. 6C). Thus, *SLC38A4* may play an important role in regulating metabolic reprogramming during CRLM.

### Immune infiltration analysis of SLC38A4

**Fig. 7:**
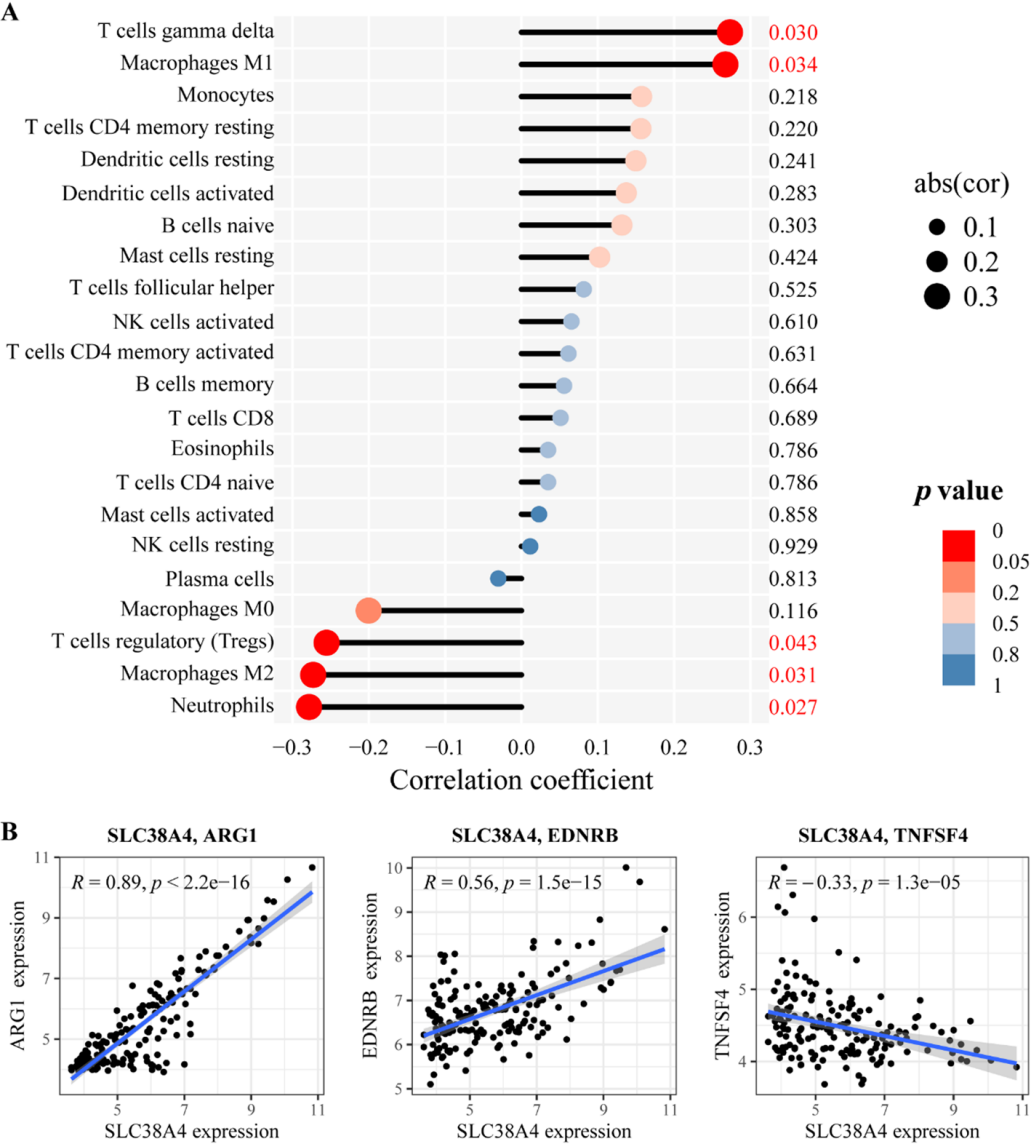
Correlation of SLC38A4 with immune cell infiltration via a lollipop map **A** and immune checkpoint genes such as ARG, EDNRB, and TNFSF4 **B**

Given the capacity of cancer cells to promote tumor growth by inhibiting immune responses in the progression of CRLM [35, 36], the role of *SLC38A4* in tumor immunity was investigated. Figure 7A illustrates the association between *SLC38A4* expression and the infiltration of 22 immune cells. The infiltration of M1 macrophages was positively linked to *SLC38A4* expression, whereas immunosuppressive cells including M2 macrophages, Treg cells, and neutrophils showed a negative association.

Furthermore, the link between *SLC38A4* and immune checkpoint genes was examined. *SLC38A4* exhibited moderate to strong positive correlations with the immunoinhibitors *ARG1* (*R* = 0.89, *p* < 2.2e-16) and *EDNRB* (*R* = 0.56, *p* = 1.5e-15). Conversely, it was negatively linked to the immunostimulator *TNFSF4* (*R* = -0.33, *p* = 1.3e-5) (Fig. 7B).

### Drug sensitivity analysis of SLC38A4

**Fig. 8:**
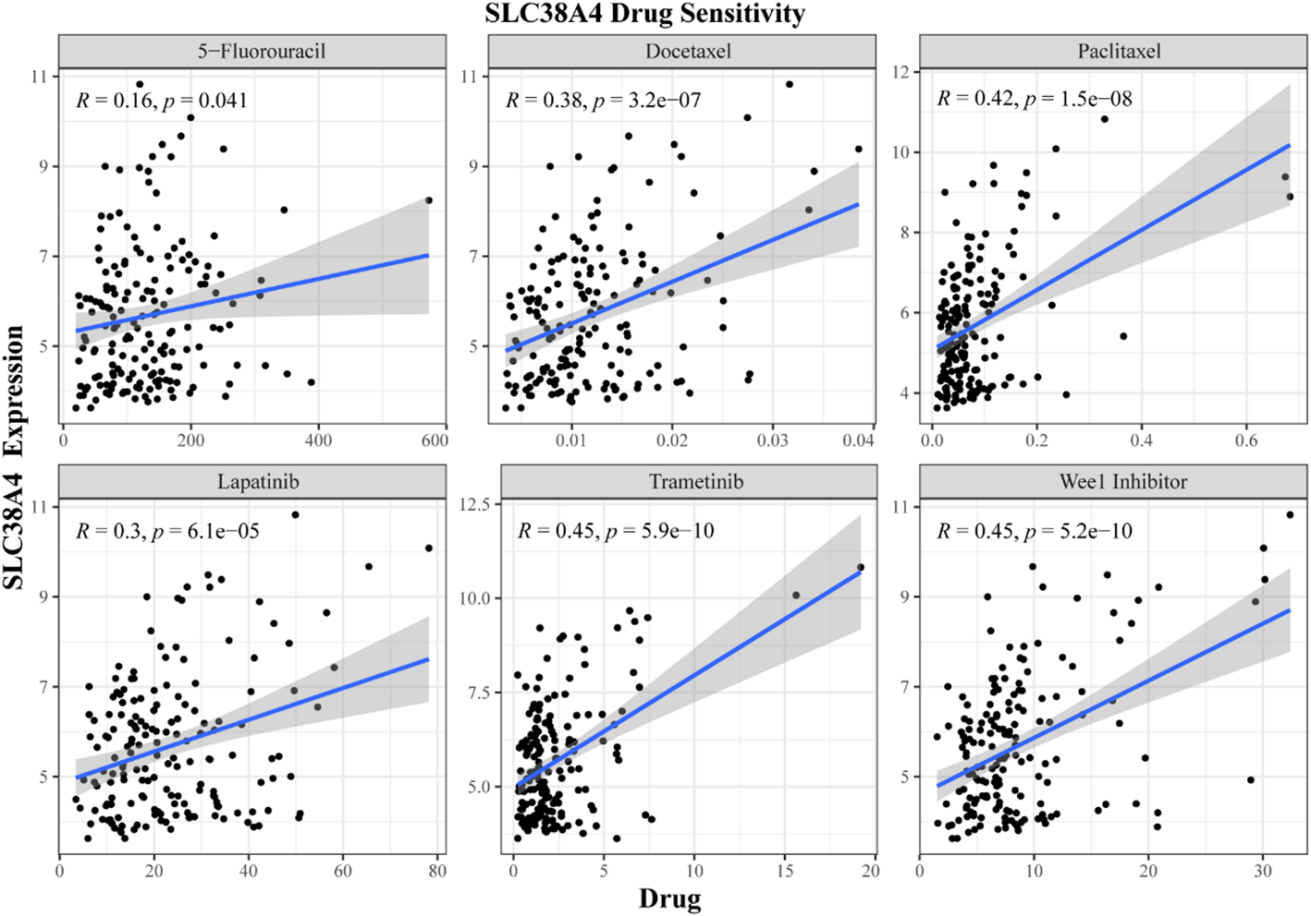
Scatter plot of the correlation between multiple anti-tumor drugs and the expression of SLC38A4

To assess the viability of *SLC38A4* as a potential treatment target in patients with CRLM, the correlation between IC50 values of anti-neoplastic agents and *SLC38A4* expression was investigated. Classical chemotherapy agents such as 5-fluorouracil, docetaxel, and paclitaxel, along with novel targeted therapies including lapatinib, trametinib, and the Wee1 inhibitor, were positively correlated with *SLC38A4* expression (Fig. 8). Supplementary Table 3 presents the correlation analysis data for all evaluated drugs.

## Discussion

CRLM is the leading site for metastasis and the main factor for mortality associated with CRC. However, therapeutic strategies for CRLM yield unsatisfactory clinical outcomes [37]. Systemic treatment, particularly targeted therapies, is limited by the lack of accurate prognostic and therapeutic biomarkers [38]. Biomarkers are central to predicting disease prognosis, monitoring disease progression and treatment efficacy, and screening appropriate treatment options [39, 40]. Nonetheless, biomarkers that truly reflect the situation of liver metastasis in CRLM are scarce. This study identified MRDEGs, conducted a prognostic analysis based on their expression in CRLM tissues, and elucidated the role of *SLC38A4* in CRLM. These findings may illuminate the prognostic significance of *SLC38A4*, positioning it as a potential biomarker for future therapeutic investigations in CRLM.

Initially, 169 MRDEGs were screened, and their prognostic impact on CRLM was investigated. To identify precise prognostic biomarkers that accurately reflect the true condition of CRLM, GSE159216 was selected as the CRLM cohort for further analysis [18]. It includes both gene expression profiles of CRLM tissues and corresponding survival data. Cox regression analyses identified nine MRDEGs, including *SLC38A4*, *RBKS*, *ASPA*, *CTH*, *ENPP1*, *TF*, *CYP39A1*, *G6PC*, and *FOM3*, as independent predictors of CRLM prognosis. Furthermore, Kaplan-Meier survival analysis suggested that only *SLC38A4* and *G6PC* serve as prognostic biomarkers associated with favorable OS. Notably, *SLC38A4* outperformed *G6PC* in prognostic power, survival prediction potential, diagnostic discrimination, and liver-specific biological relevance. Given these advantages, *SLC38A4* was selected as the primary focus for subsequent investigation.

The differential expression of SLC38A4 between CRLM and NL was validated through GEO datasets, qRT-PCR, WB, and IHC experiments. The *SLC38A4*-encoded protein is predominantly expressed in human liver tissues [34], consistent with our findings. Within the tumor, SLC38A4 exhibited predominantly membranous and cytoplasmic staining, reflecting its role in transporting amino acids [22]. Furthermore, the expression of SLC38A4 was downregulated in CRLM tissues compared to NL tissues. These findings have not been previously reported in CRLM. Additionally, SLC38A4 expression was silenced in HCC compared to NL tissues [24, 25], aligning with our findings.

To obtain deeper insights into the role of *SLC38A4* and its interacting partners in CRLM, GO and KEGG analyses were conducted. *SLC38A4* and its correlated genes predominantly participate in diverse metabolic processes and pathways. Furthermore, GSEA findings suggested that high expression of *SLC38A4* is linked to numerous metabolic pathways for various substrates. Abnormalities in cancer metabolism are one of the key cancer hallmarks [41]. Metabolic reprogramming contributes to tumor initiation, progression, and metastasis [42, 43]. Moreover, metabolic contributions from metastatic sites are important drivers of colonization and outgrowth by disseminated cancer cells [44]. For CRLM, hepatic metastatic CRC cells can reprogram metabolic pathways through substances, such as *ALDOB* and exosomal *HSPC111*, promoting colonization and outgrowth [45, 46]. Our findings indicated that *SLC38A4* may mediate metabolic reprogramming in the liver, consistent with previous studies [44–46]. Contrarily, *SLC38A4* may serve as a suppressor, inhibiting CRLM progression. Additional mechanistic investigations are required to determine the function of *SLC38A4* in CRLM.

The TME consists of various types of cells that interact with tumor cells [47]. It is central to tumor genesis, progression, invasion, and metastasis [48–50]. Modulating the balance between tumor-promoting and tumor-inhibiting immune cells within the TME holds promise for cancer treatment. Our study indicated that the expression of *SLC38A4* positively correlates with M1 macrophage infiltration but negatively correlates with M2 macrophages, Treg cells, and neutrophils. M1 macrophages exert anti-tumor effects, while M2 macrophages promote tumor growth [51, 52]. Treg cells suppress tumor-specific immune responses [53], and neutrophils promote the proliferation and metastasis of cancer cells via multiple pathways [54]. Additionally, the links between *SLC38A4* and immune checkpoint genes were investigated. *SLC38A4* was positively associated with *ARG1* and *EDNRB*, while it was negatively associated with *TNFSF4*. *ARG1* promotes tumor immune evasion by depleting L-arginine in the TME, leading to T cell dysfunction [55]. *EDNRB* functions as an immune regulator within the TME, contributing to immune evasion and angiogenesis via tumor-associated macrophages [56]. Paradoxically, EDNRB overexpression inhibits proliferation and migration of prostate cancer cells through activation of the cGMP-PKG pathway [57]. *TNFSF4* (*OX40L*) is a critical co-stimulatory molecule for T cell activation and adaptive immunity. *OX40*/*OX40L* signaling enhances T cell functions, counteracts Treg suppression, and boosts anti-tumor immunity [58]. Collectively, *SLC38A4* is significantly linked to the infiltration of immune cells and key immune checkpoint genes, suggesting its potential role in modulating the immune landscape of the TME.

Drug resistance remains a primary contributor to deaths caused by cancer [59]. Advances in genome-wide screening methods have elucidated the evolution of tumor responses to therapeutic interventions, providing novel insights to mitigate drug resistance and improve treatment efficacy [60]. Various anti-tumor drugs, including conventional chemotherapy agents like 5-fluorouracil, docetaxel, and paclitaxel, as well as novel targeted therapies such as lapatinib, trametinib, and Wee1 inhibitor, have demonstrated therapeutic efficacy against various malignancies, including CRC and CRLM [61–63]. Our research demonstrated that the effectiveness of these agents was positively connected to the upregulation of *SLC38A4*. Therefore, targeting *SLC38A4* could potentially enhance the efficacy of these drugs in treating CRLM. However, the mechanisms behind these correlations warrant further investigation.

To our knowledge, this research is the first to investigate the function of *SLC38A4* in patients with CRLM. *SLC38A4* acts as a favorable prognostic biomarker with mechanisms connected to metabolic reprogramming and immune cell infiltration. Therefore, *SLC38A4* may be a potential biomarker for future therapeutic investigation. However, the research has specific limitations. Firstly, our analysis was limited to one CRLM dataset (GSE159216), as it was the only resource meeting our criteria: transcriptomic profiles from CRLM tissues and matched survival data. To offer additional context, we validated findings in primary CRC using the TCGA-COADREAD dataset, where high *SLC38A4* expression similarly showed favorable prognostic significance (Fig. S3). While biological differences between primary and liver metastatic lesions exist, this reinforces *SLC38A4*’s prognostic implication. Future studies will include additional CRLM cohorts for further validation of our findings. Secondly, this bioinformatics-driven research provides a preliminary understanding of *SLC38A4*’s role in CRLM; however, its detailed molecular mechanisms are still unclear and require further experimental functional verification (e.g., knockdown or overexpression). This issue will be addressed in our upcoming research. Lastly, the absence of multiple testing correction in immune cell correlations and drug sensitivity analyses may increase the risk of false positives. We will validate the findings through experimental approaches.

## Conclusion

The research indicated the significance of *SLC38A4* in the prognosis and biological aspects of CRLM. *SLC38A4* is related to a favorable prognosis in CRLM patients, potentially through its impact on immune infiltration. Thus, *SLC38A4* may function as a prognostic biomarker and a potential candidate for future therapeutic investigation, providing a novel perspective on precision oncology for patients with CRLM.

## Supplementary Information

Below is the link to the electronic supplementary material.

Supplementary Material 1.

Supplementary Material 2.

Supplementary Material 3.

Supplementary Material 4.

Supplementary Material 5.

Supplementary Material 6.

## Data Availability

The gene expression data analyzed in this study were sourced from public repositories. The datasets can be accessed via the Gene Expression Omnibus (GEO) under accession numbers GSE38174, GSE41258, and GSE159216 (https:/www.ncbi.nlm.nih.gov/geo), and The Cancer Genome Atlas (TCGA) colon and rectal cancer (TCGA-COADREAD) project ( https://portal.gdc.cancer.gov ).

## Abbreviations

CRLM: Colorectal liver metastases
NL: Normal liver
CRC: Colorectal cancer
HCC: Hepatocellular carcinoma
IHC: Immunohistochemistry
OS: Overall survival
GEO: Gene Expression Omnibus
DEGs: Differentially expressed genes
MRGs: Metabolism-related genes
MRDEGs: Metabolism-related differentially expressed genes
WGCNA: Weighted gene co-expression network analysis
GO: Gene Ontology
MF: Molecular function
CC: Cellular components
BP: Biological processes
GSEA: Gene set enrichment analysis
KEGG: Kyoto Encyclopedia of Genes and Genomes
TME: Tumor microenvironment
