# Supplementary figures and images for: SLC38A4 as a prognostic biomarker and correlated with immune infiltration in colorectal liver metastasis
Source: Discov Oncol. 2025 Sep 2;16:1675. doi: 10.1007/s12672-025-03509-9 (PMC12405119; doi:10.1007/s12672-025-03509-9)

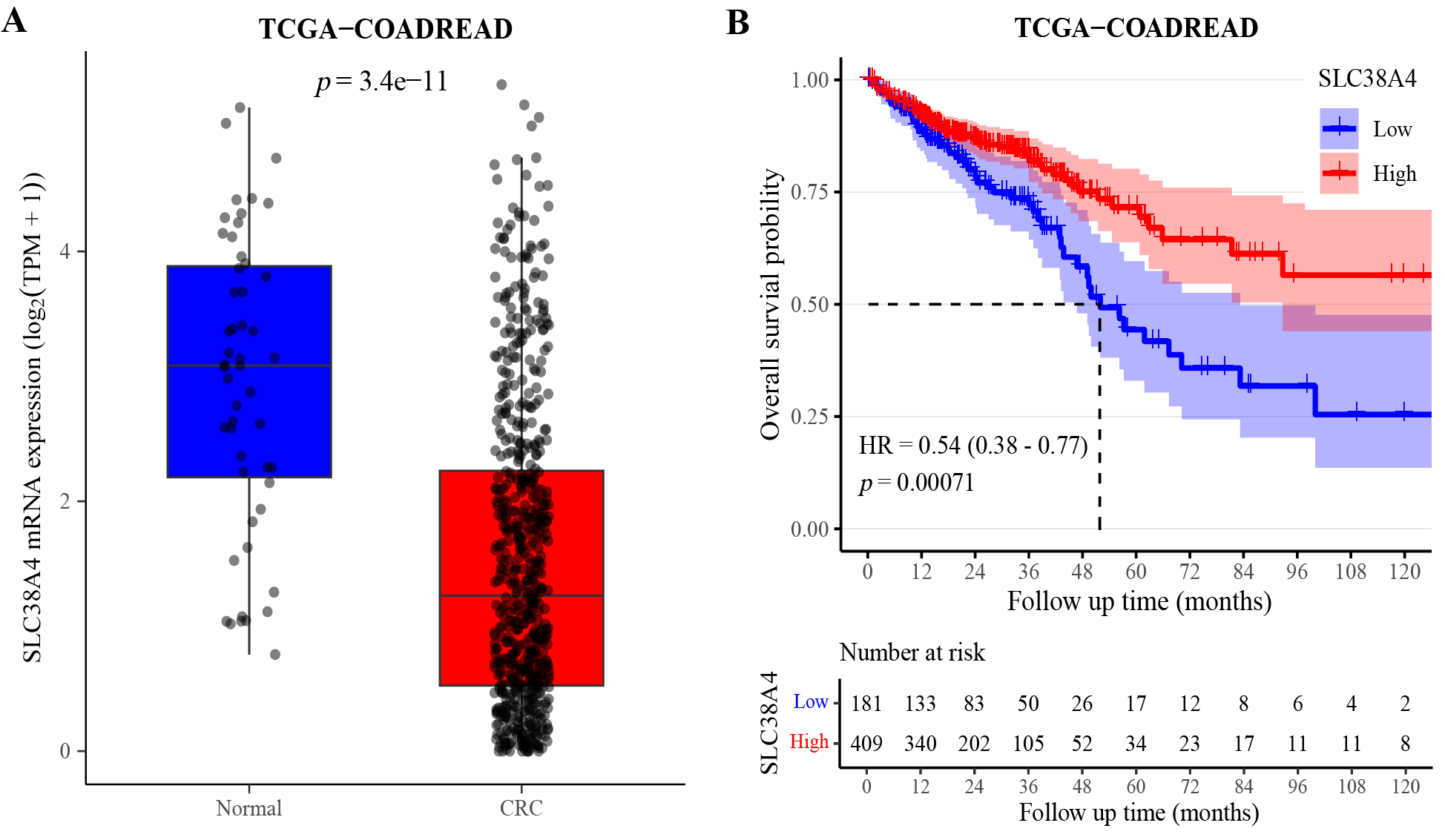

Supplement: Supplementary file 4 — Supplementary Material 4. [file 12672_2025_3509_MOESM4_ESM.tif]

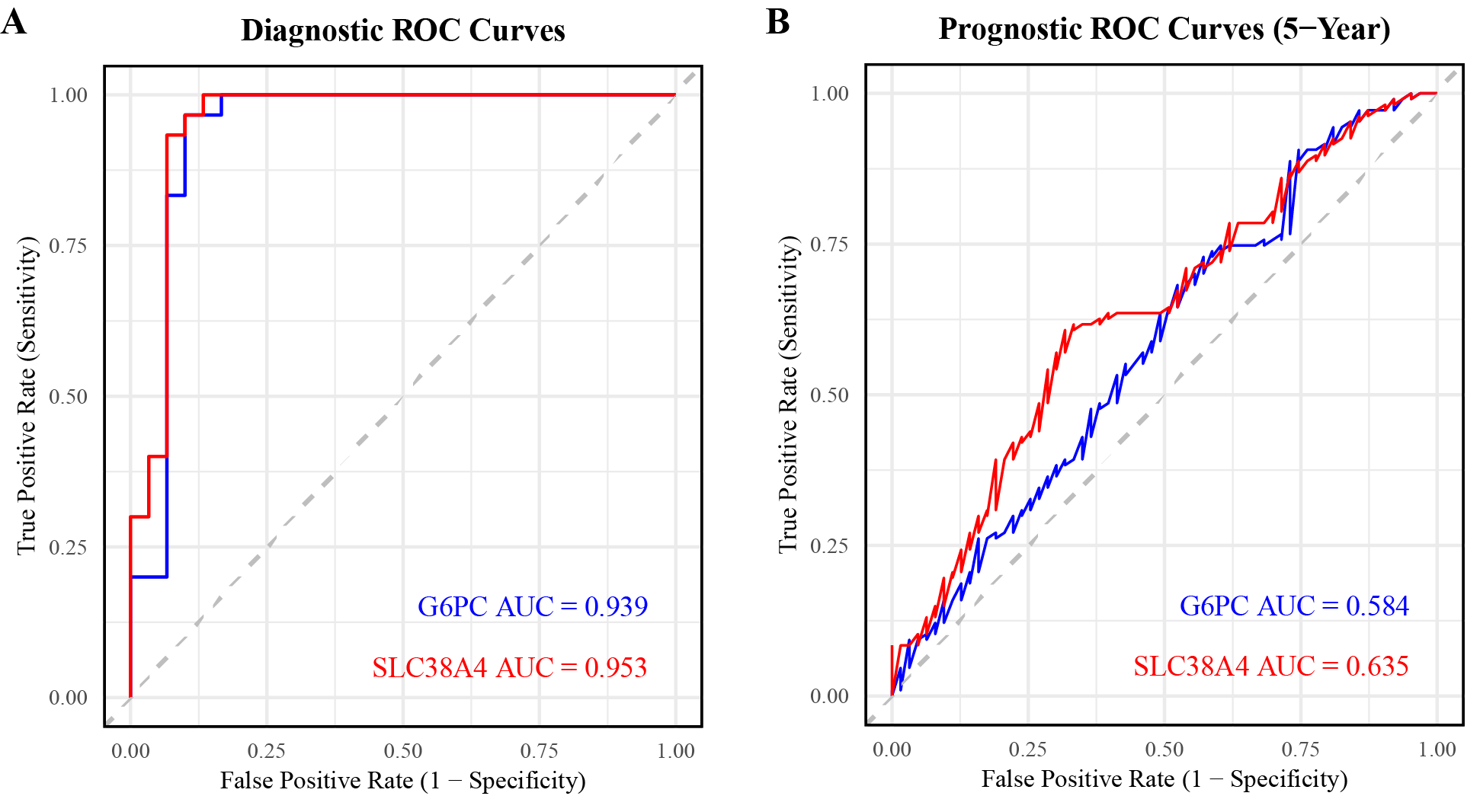

Supplement: Supplementary file 5 — Supplementary Material 5. [file 12672_2025_3509_MOESM5_ESM.tif]

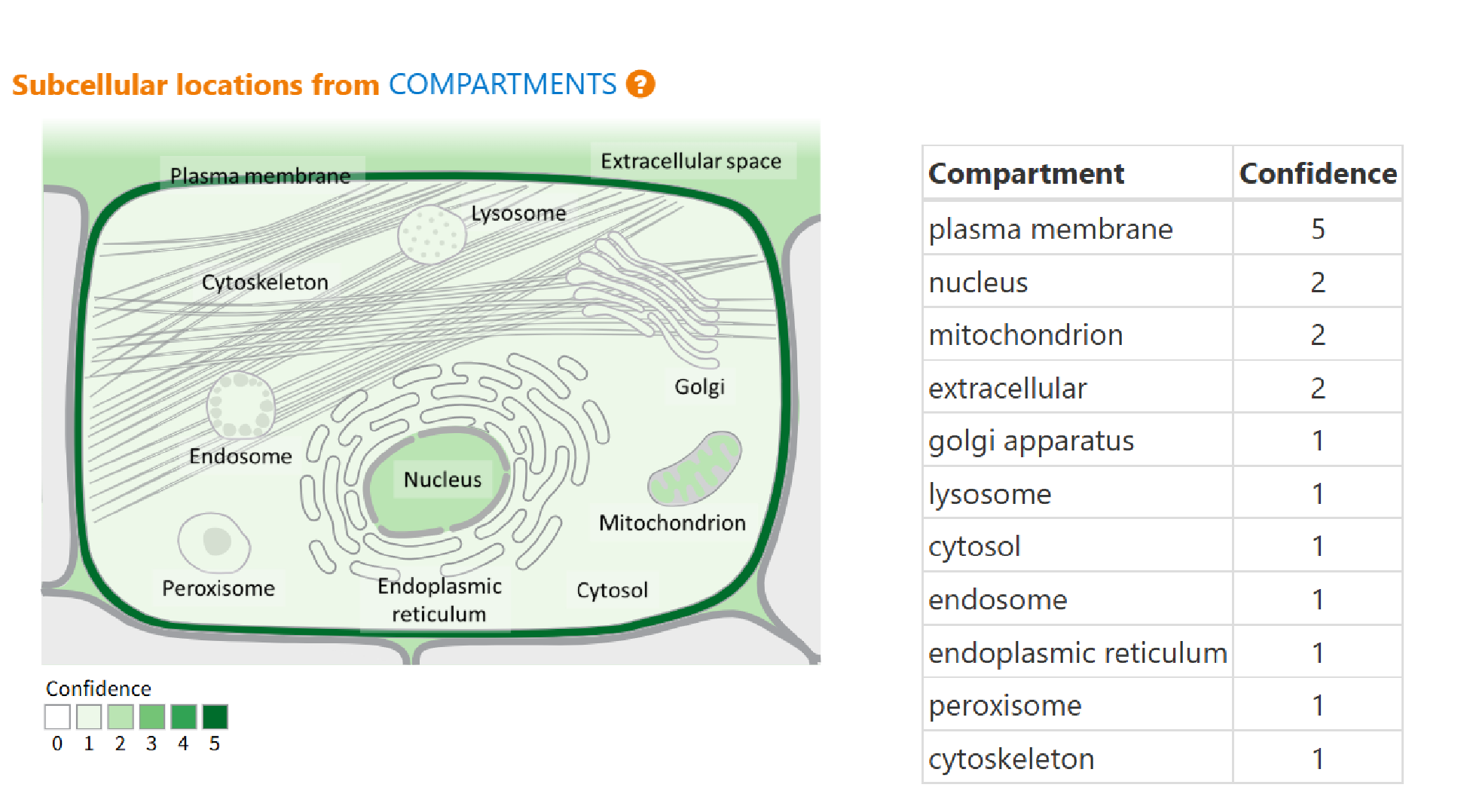

Supplement: Supplementary file 6 — Supplementary Material 6. [file 12672_2025_3509_MOESM6_ESM.tif]
